# Camel whey protein enhances diabetic wound healing in a streptozotocin-induced diabetic mouse model: the critical role of β-Defensin-1, -2 and -3

**DOI:** 10.1186/1476-511X-12-46

**Published:** 2013-04-01

**Authors:** Gamal Badr

**Affiliations:** 1 Present address: Princess Al-Johara Al-Ibrahim Center for Cancer Research, Prostate Cancer Research Chair, College of Medicine, King Saud University, P.O. Box 7805, Riyadh, 11472, Saudi Arabia; 2 Permanent address: Zoology Department, Faculty of Science, Assiut University, Assiut, 71516, Egypt

**Keywords:** Diabetes, Defensins, Pro-inflammatory cytokines, Whey protein, Wound healing

## Abstract

**Background:**

Delayed wound healing is considered one of the most serious diabetes-associated complications. The presence of replicating organisms such as bacteria within a diabetic’s wound is considered one of the most important factors that impair cutaneous wound healing and the potential cellular and/or molecular mechanisms that are involved in the healing process. Defensins, which are anti-microbial peptides, have potent bactericidal activity against a wide spectrum of the bacterial and fungal organisms that are commonly responsible for wound infections. We recently demonstrated that camel whey proteins (WPs) expedite the healing of diabetic wounds by enhancing the immune response of wounded tissue cells and by alleviating some of the diabetic complications.

**Methods:**

In the present study, we investigated the effects of WP supplementation on the mRNA and protein expression levels of β-defensin-1 (BD-1), 2 and 3 and subsequently on the wound healing process in a streptozotocin (STZ)-induced diabetic mouse model. In this study, three groups of mice were used (10 mice per group): group 1, the non-diabetic mice (control); group 2, the diabetic mice; and group 3, the diabetic mice that received a daily supplement of undenatured WP (100 mg/kg of body weight) via oral gavage for 1 month.

**Results:**

Compared with the non-diabetic control mice, the diabetic mice exhibited delayed wound closure that was characterized by a reduction in hydroxyproline content (indicator of collagen deposition), a marked elevation in free radical levels and a prolonged elevation in the levels of inflammatory cytokines, including interleukin-6 (IL-6), transforming growth factor-beta (TGF-β) and tumor necrosis factor-alpha (TNF-α). Interestingly, compared with the diabetic mice that did not receive WP supplementation, the diabetic mice with WP had an accelerated closure and healing process of their wounds. The WP supplementation also decreased their levels of free radicals and restored their hydroxyproline content; proinflammatory cytokine levels; and expression of BD-1, 2 and 3 in the wounded tissue.

**Conclusion:**

WP supplementation may be beneficial for improving the healing and closure of diabetic wounds.

## Background

Impaired wound healing, a common complication of diabetes mellitus, is characterized by diminished collagen production and impaired angiogenesis [1]. These complications are caused by the action of released free radicals [2], which damage multiple cellular components, such as lipids, proteins and DNA. Furthermore, multiple factors in the diabetic wound, including increased apoptosis, decreased vascular recovery, an aberrant inflammatory response and delayed cellular turnover, contribute to impaired wound healing [3]. Additionally, the production of reactive oxygen species (ROS) by inflammatory cells and other cell types in the wound is required for defense against invading bacteria. Moreover, at physiological levels, ROS are also important regulators of various intracellular signaling pathways [4]. Oxidative stress is an important pathogenic factor in diabetic wound complications and affects cell replication and life span. Intracellular glutathione (GSH) can normalize skin cell functions that are disrupted by *in vitro* cell growth under hyperglycemic conditions [5]. Although ROS plays crucial roles in cell signaling and in the immune response, higher levels of ROS cause oxidative stress during wound healing. Therefore, regulating oxidative stress and the inflammatory response is an important factor in cutaneous wound healing. Wound healing can be defined as a complex, multi-stage process that involves distinct phases: inflammation, the formation of granulation tissue, the production of new structures and tissue remodeling [6]. Moreover, many factors can interfere with one or more phases of this process and thus may affect wound healing by causing improper or impaired tissue repair. Collagen is the predominant extracellular protein in the granulation tissue of a healing wound. In addition, the synthesis of collagen rapidly increases in the wound area soon after an injury, and this increase in collagen provides strength and integrity to the tissue matrix. The measurement of hydroxyproline, which is produced by the breakdown of collagen, has been used as an index of collagen turnover [7]. The cytokines and chemokines secreted by skin-resident cells (keratinocytes, fibroblasts and endothelial cells) and by inflammatory cells are involved in wound healing. Proinflammatory cytokines, including interleukins 1α and 1β (IL-1α and IL-1β), IL-6 and tumor necrosis factor alpha (TNF-α), play important roles in wound repair, such as the stimulation of keratinocyte and fibroblast proliferation, synthesis and breakdown of extracellular matrix proteins, fibroblast chemotaxis and regulation of the immune response [8]. In addition, recent reports have indicated that the dysregulation of TNF-α impairs the healing of diabetic wounds and that this dysregulation may involve enhanced apoptosis and decreased proliferation of fibroblasts [9]. Previous studies have demonstrated that transforming growth factor-β (TGF-β) plays critical roles in wound repair. This cytokine functions in leukocyte chemotaxis, fibroblast and smooth muscle cell mitogenesis and extracellular matrix deposition during granulation tissue formation [8,10]. One common denominator associated with the regulation of wound healing events is human β-defensin 2 (BD-2). Skin wounding induces cutaneous BD-2 expression, and diabetic wounds have been associated with inadequate human β-defensin expression [11]. Human BD-2 may also participate in other aspects of innate immunity because it chemoattracts monocytes and immature dendritic cells [12]. In burned skin, human β-defensin 1 (BD-1) is expressed by the dermal glands, including hair shafts. Moreover, human BD-2 and 3 have been found in the remaining keratin layers and glands of the lower dermis [13]. In a recently published study, burn wounds exhibited a moderately lower expression of BD-1 than healthy tissues [14].

The improvement in immune function using dietary antioxidants can play an important role in the prevention of many human diseases and diabetic complications. Camel whey proteins (WPs) include a heterogeneous group of proteins, including serum albumin, α-lactalbumin, immunoglobulin, lactophorin and peptidoglycan recognition protein [15]. Dietary whey supplementations may improve wound healing by increasing GSH synthesis and cellular antioxidant defense [16]. Therefore, WPs may be a therapeutic tool for the treatment of oxidative stress-associated diseases [17]. The oral administration of an undenatured WP increases the GSH levels of severa GSH-deficient patients, including those with advanced human immunodeficiency virus (HIV) infection [18]. Recent studies have indicated that whey increases the antioxidant activity in the body, combats fatigue and inflammation, hastens healing, improves stamina and may discourage related infections due to the immune system-enhancing and natural antibiotic properties of its components [19,20]. Nevertheless, few studies have investigated the influence of WPs on wound healing. The aim of this study was to investigate the effects of WP supplementation on the mRNA and protein expression levels of BD-1, 2 and 3 and subsequently to observe the effects of WP on the wound healing process in a streptozotocin (STZ)-induced diabetic mouse model.

## Materials and methods

### Preparation of WPs

Raw camel milk was collected from healthy female camels from Assiut Governorate, Egypt. The milk was then centrifuged to remove the cream. The skim milk obtained was acidified to pH 4.3 using 1 N HCl at room temperature and centrifuged at 10,000 × *g* for 10 min to precipitate the casein. The resultant whey, which contained the WPs, was saturated with ammonium sulfate to a final saturation of 80% to precipitate the WPs. The precipitated WPs were dialyzed against 20 volumes of distilled water for 48 h using a molecular-porous membrane with a molecular weight cut off (MWCO) of 6,000–8,000 kDa. The dialysate containing the undenatured WPs was freeze-dried and refrigerated until use.

### Chemicals

STZ was obtained from Sigma Chemicals Co. (St. Louis, MO, USA). STZ was dissolved in cold 0.01 M citrate buffer (pH 4.50) and was always freshly prepared for immediate use (within 5 min).

### Animals and experimental design

A total of 30 sexually mature 12-week-old male Swiss Webster (SW) mice, each of which weighed between 25 and 30 g, were obtained from the Central Animal House of the Faculty of Pharmacy at King Saud University. All of the animal procedures were conducted in accordance with the standards set forth in the Guidelines for the Care and Use of Experimental Animals by the Committee for the Purpose of Control and Supervision of Experiments on Animals (CPCSEA) and the National Institutes of Health (NIH). The study protocol was approved by the Animal Ethics Committee of King Saud University in accordance with the principles of Declaration of Helsinki. All of the animals were allowed to acclimate to the metal cages inside a well-ventilated room for 2 weeks prior to experimentation. The animals were maintained under standard laboratory conditions (23°C, 60–70% relative humidity and a 12-h light/dark cycle), fed a diet of standard commercial pellets and given water ad libitum. All of the mice were fasted for 20 h before diabetes induction. The mice (*n* = 20) were rendered diabetic with an intraperitoneal (i.p.) injection of a single dose of STZ (60 mg/kg of body weight) in 0.01 M citrate buffer (pH 4.5) [21]. The mice in the control group (*n* = 10) were injected with the vehicle (0.01 M citrate buffer, pH 4.5). The animals were divided into three experimental groups: group 1 was the control non-diabetic mice that received a daily supplement of 250 μl of distilled water through oral gavage for one month; group 2 was the diabetic mice that received 250 μl of distilled water daily through oral gavage for one month; and group 3 was the diabetic mice that were daily supplemented with undenatured WP (100 mg/kg of body weight) dissolved in 250 μl of distilled water via oral gavage for one month. Therefore, the volume of the daily supplement received by each mouse in the three groups was constant and did not exceed 250 μl. The optimal dose of WP was determined in our laboratory based on the LD_50_ and several established measured parameters.

### Excisional wound preparation and macroscopic examination

Following the diabetes induction in groups 2 and 3, the mice in each group were wounded at the age of 12 weeks. The wounding of the mice was performed as described previously [22]. Briefly, the mice were anaesthetized with a single i.p. injection of ketamine (80 mg/kg of body weight) and xylazine (10 mg/kg of body weight). The hair on the back of each mouse was cut, and the back was subsequently wiped with 70% ethanol. Six full-thickness wounds (5 mm in diameter, 3–4 mm apart) were made on the back of each mouse by excising the skin and the underlying panniculus carnosus. The wounds were allowed to form a scab. Skin biopsy specimens were obtained from the animals 1, 4, 7, 10 and 13 days post wound injury. At each time point, an area that included the scab, the complete epithelial and dermal compartments of the wound margins, the granulation tissue and parts of the adjacent muscle and subcutaneous fat tissue was excised from each individual wound. As a control, a similar amount of skin was collected from the backs of non-wounded normal mice. Each wound site was digitally photographed at the indicated time points to determine the wound area. Changes in the wound areas were expressed as the percentage of the initial wound areas. At the indicated time points, tissue from two wounds from each of 10 animals (for a total of 20 wounds) was collected for RNA analysis.

### Histopathological studies

A specimen sample was isolated from each group of mice on day 13 after wounding for histopathological examination. The skin specimens were immediately fixed in 10% (v/v) neutral buffered formalin, and the fixative solution was replaced every 2 days until the tissues hardened. Each specimen was embedded in a paraffin block, and thin sections (3 μm) were prepared and stained with hematoxylin and eosin (H&E) for general morphological observations.

### Blood analysis

The blood glucose levels were determined using an AccuTrend sensor (Roche Biochemicals, Mannheim, Germany). The serum insulin level was analyzed by Luminex (Biotrend, Düsseldorf, Germany) according to the manufacturer’s instructions.

### Quantification of biochemical parameters in wound tissue

#### Measurement of the hydroxyproline content

After drying for 24 h at 120°C, the amount of hydroxyproline, which is a major constituent of collagen in skin wound sites, was measured to index the collagen accumulation at the wound site, as previously described [23]. The hydroxyproline contents were expressed as the amount (mg) per wound.

#### Measurement of the cytokine levels

A 2.0-mm punch biopsy at the wound site was harvested and frozen in liquid nitrogen. The specimens were homogenized in cytoplasmic lysis buffer containing protease inhibitors (Roche Diagnostics), disrupted using Fast Prep (Q-Biogene, Solon, OH, USA) and centrifuged at 5000 × *g* for 10 min. The protein concentration in each lysate was determined using the bicinchoninic acid (BCA) protein assay kit (Pierce). The levels of IL-6, TGF-β and TNF-α in the supernatants were determined using a commercial ELISA kit (R&D Systems, France) according to the manufacturer’s instructions. For each sample, the data were expressed as the amount of the target molecule (picograms) divided by the amount of total protein (milligrams).

#### Measurement of the free radical levels

The tissue lysates were also used to determine the ROS levels using 2,7-dichlorodihydrofluorescein diacetate (H2DCF-DA; Beyotime Institute of Biotechnology, Haimen, China). The hydroperoxide levels were evaluated using a free radical analytical system (FRAS 2, Iram, Parma, Italy), which is a colorimetric test that takes advantage of the ability of hydroperoxide to generate free radicals after reacting with transitional metals.

#### Extraction of total RNA and RT-PCR analysis

Total RNA was isolated from the wounded skin samples using the TRIzol (Invitrogen Life Technologies, France) reagent according to the manufacturer’s instructions. Before reverse transcription, the RNA was treated with RNase-free DNase I following the manufacturer’s protocol. Three micrograms of the total RNA was used for the synthesis of cDNA using a Superscript III RT kit (Invitrogen Life Technologies, France). Unique primer sets for mouse BD-1, 2 and 3 and β-actin were designed (Table 1) based on sequences deposited in the National Center for Biotechnology Information and were synthesized by Invitrogen Life Technologies. The PCR was performed using 1 μl of cDNA in a reaction mix with *Taq* polymerase (Invitrogen Life Technologies). The PCR was found to be linear between 20 and 35 cycles, and the PCR conditions were optimized to allow a semi-quantitative comparison of the results. The digital images of the bands separated on ethidium bromide-stained agarose gels were quantified using the NIH Image Analysis Software. The intensity of each primer product was normalized to the intensity of the β-actin primer product and expressed relative to the levels found in the injured skin of the control non-diabetic mice.

**Table 1:** Sequences of primers used for RT-PCR

**Transcript**	**Sequence**	**Product size (bp)**
β-defensin-1	(F) 5´-ATGAAAACTCATTACTTTCTCCTGG-3´	216
	(R) 5´-ACTACTGTCAGCTCTTACAACA-3´	
β-defensin-2	(F) 5´-ATACGAAGCAGAACTTGACCACTG-3´	131
	(R) 5´-AATCATTTCATGTACTTGCAACAGG-3´	
β-defensin-3	(F) 5´-GCGAATTCATGAGGATCCATTACCTTCTCTT-3´	209
	(R) 5´-GCCTCGAGCTATTTTCTCTTGCAGCATTTG-3´	
β-actin	(F) 5´-CCATGGATGACGATATCGCTGC-3´	669
	(R) 5´-GCTTCTCTTTGATGTCACGCACG-3´	

(F), Forward primer; (R), reverse primer.

### Western blot analysis

The skin and wound tissue biopsies were homogenized in lysis buffer (1% Triton X-100, 137 mM NaCl, 10% glycerol, 1 mM dithiothreitol, 10 mM NaF, 2 mM Na3VaO4, 5 mM ethylenediaminetetraacetic acid, 1 mM phenylmethylsulfonylfluoride, 5 ng/ml aprotinin, 5 ng/ml leupeptin and 20 mM Tris/HCl, pH 8.0), and the lysates were prepared as previously described [24]. Fifty micrograms of total protein from the skin lysates was analyzed using SDS polyacrylamide gel electrophoresis (SDS-PAGE) and western blot analysis. The proteins were transferred to a nitrocellulose membrane (0.2 μm, Amersham Hybond ECL, GE Healthcare, Freiburg, Germany), blocked for 1 h in blocking buffer (5% (wt/vol) BSA in PBS + 0.05% Tween), incubated overnight at 4°C in 3% (wt/vol) BSA in PBS + 0.05% Tween containing either 1:250 anti-mBD-1, 1:250 anti-mBD-3 and 1:1000 mBD-3 antibodies (all from Santa Cruz Biotechnology, Santa Cruz, CA, USA) or 1:1000 anti-β actin antibody (Sigma). The membrane was washed five times with PBS + 0.05% Tween for 5 minutes and incubated for 1 h in 3% (wt/vol) BSA in PBS + 0.05% Tween containing a 1:5000 dilution of goat anti-rabbit or rabbit anti-goat IgG conjugated to horseradish peroxidase (HRP, Dianova, Hamburg, Germany). After five additional washes, the proteins were visualized using an enhanced chemiluminescence (ECL, SuperSignal West Pico chemiluminescent substrate; Perbio, Bezons, France) detection system. The ECL signal was detected on Hyperfilm ECL. To quantify the band intensities, the films were scanned, saved as TIFF files and analyzed using the NIH Image J software.

### Statistical analysis

The data were first tested for normality (using the Anderson-Darling test) and for variance homogeneity prior to any further statistical analyses. The data were normally distributed and were expressed as the mean ± SEM (standard error of the mean). The differences between the groups were analyzed by one-way analysis of variance (for more than two groups) followed by Tukey’s post-test using the SPSS software (version 17). The data are expressed as the mean ± SEM. The differences were considered statistically significant at ^*^P< 0.05 for diabetic vs. control; ^**+**^P< 0.05 for diabetic + WP vs. control; or ^**#**^P< 0.05 for diabetic + WP vs. diabetic.

## Results

### Supplementation with camel WP improved wound closure in diabetic mice

**Figure 1:**
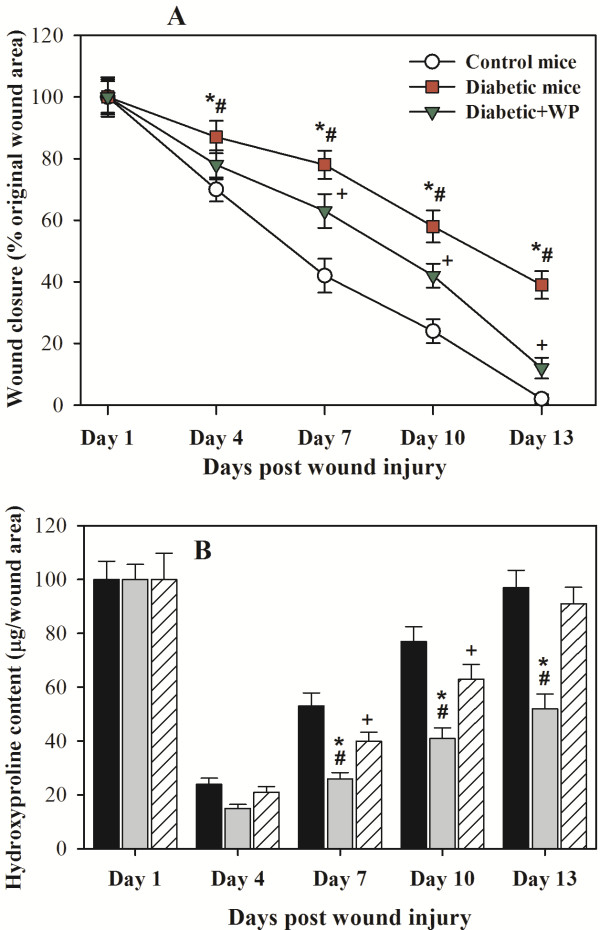
**Macroscopic changes in the skin excisional wound sites that determine wound closure.** (**A**) The changes in the percentage of wound closure at each time point after wound injury were measured relative to the original wound area on day 1. The accumulated data on the changes in wound closure from ten individual mice in each group are shown. The data are presented as the mean ± SEM. ^*^P < 0.05, diabetic vs. control; ^**+**^P < 0.05, diabetic + WP vs. control; ^**#**^P < 0.05, diabetic + WP vs. diabetic. (**B**) The hydroxyproline content, which was used as an index of the collagen accumulation at the wound site, was determined. The data are presented as the mean ± SEM. ^*^P < 0.05, diabetic vs. control; ^**+**^P < 0.05, diabetic + WP vs. control; ^**#**^P < 0.05, diabetic + WP vs. diabetic.

We first evaluated the macroscopic changes in the skin excisional wound sites of the control mice, diabetic mice and diabetic mice supplemented with WP. The day 1 pictures were obtained immediately after the injury. We observed that the wound sites of the mice in all of the experimental groups exhibited similar morphology on day 1 post injury, whereas the wounds of the control and WP-supplemented diabetic groups were almost similarly closed by day 13 post injury. By contrast, the diabetic mice exhibited delayed wound closure. Data from 10 individual mice in each group were used to determine the changes in the percentage of wound closure at each time point compared with the original wound area (Figure 1A). The diabetic mice supplemented with camel WP had accelerated wound closure and thus the eventual healing compared with the diabetic mice, which exhibited delayed wound closure.

Because hydroxyproline is a major constituent found almost exclusively in collagen, we used its content as an indicator of the amount of collagen type I at the wound sites. The accumulated data from 10 individual mice in each group demonstrated that compared with the control mice, the diabetic mice had significantly lower hydroxyproline content and decreased wound closure (Figure 1B). Compared with the control mice, the diabetic mice exhibited less collagen accumulation at the wound sites, which confirmed the presence of a delayed healing process. However, compared with the diabetic mice, the diabetic mice supplemented with camel WP exhibited a significant restoration of their hydroxyproline content. These results suggest that collagen production was enhanced through the oral administration of WP.

### Effect of WP supplementation on the histopathological features of the healed wounds of diabetic mice

**Figure 2:**
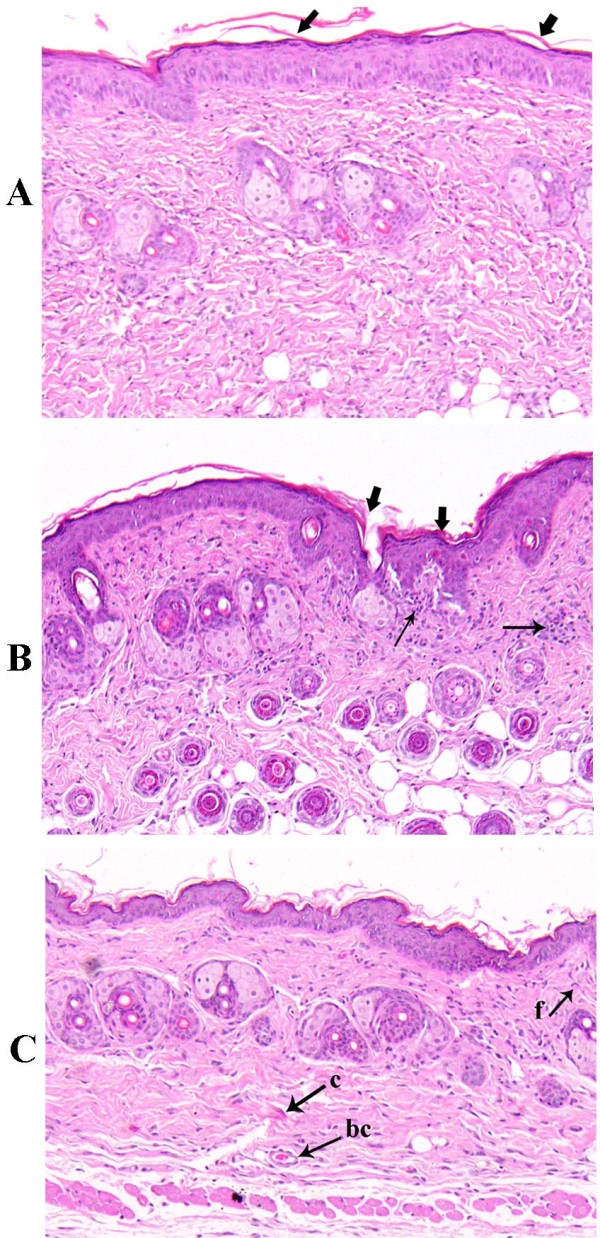
**Histological examination of healed wound sections stained with H&E.** The photomicrographs show the healed wound sections isolated from mice in the (**A**) control, (**B**) diabetic and (**C**) diabetic +WP group on day 13 after wounding. The photomicrographs were obtained at a magnification of ×200. Abbreviations: bc, blood capillaries; C, collagen fibers; f, fibroblast.

The histopathological characteristics of the healed wounds on day 13 after wounding are shown in Figure 1. The control group exhibited good wound healing with complete re-epithelialization and well-formed connective tissue (Figure 2A). Conversely, the tissue obtained from the diabetic group exhibited disorganized fibroblasts, an absence of collagen fiber deposition and the infiltration of inflammatory cells (arrows) (Figure 2B). Interestingly, a greater degree of tissue regeneration was observed in the diabetic + WP group, as demonstrated by complete epithelization, significantly higher collagen deposition and the presence of granulation tissues (Figure 2C).

### WP treatment of diabetic mice decreased the levels of free radicals in wounded tissue

To optimize all of the parameters and conditions of the animal models during the experiments, the blood glucose and insulin levels of the mice in the three groups were monitored throughout the indicated time points post wound injury (Table 2). We observed that the blood glucose levels of the WP-treated diabetic mice were significantly lower than those of the diabetic mice and were higher than those of the control mice. By contrast, the WP-treated diabetic mice had significantly higher levels of insulin than the non-treated diabetic mice throughout the wound healing period. The control and WP-treated diabetic mice had significantly lower hydroperoxide and ROS levels in the wound tissue than the diabetic mice (Table 2).

**Table 2:** Effect of WP supplementation on the levels of free radicals in the wound tissues of diabetic mice

		**Blood parameters**		**Wound tissue parameters**	
**Day post injury**	**Groups**	**Glucose**	**Insulin**	**ROS**	**Hydroperoxide**
		**(mg/dl)**	**(ng/ml)**	**(μmol/ml)**	**(mg/100ml)**
**Day 1**	**Control**	137±13	5.1±0.48	41±5.1	23±2.2
	**Diabetic**	411±37 *#	1.7±0.15 *#	92±8.4 *#	59.1±4.6 *#
	**Diabetic + WP**	287±27 **+**	3.1±0.29 +	68±7.1 +	33.4±4.2 +
**Day 4**	**Control**	143±14.4	6.4±0.61	58±6.2	17±2.1
	**Diabetic**	393±33 *#	1.4±0.3 *#	117±12 *#	67±5.3 *#
	**Diabetic + WP**	279±28 **+**	2.9±0.25 +	79±8.1 +	37.6±4.1 +
**Day 7**	**Control**	111±13	5.7±0.5	49.8±5.1	27.1±2.9
	**Diabetic**	376±33 *#	1.1±0.22 *#	132±14 *#	59.8±5.5 *#
	**Diabetic + WP**	267±24.8 **+**	3.4±0.3 +	71.7±8 +	42.1±4.1 +
**Day 10**	**Control**	99±10.4	7.1±0.75	61.5±5.6	19.9±1.7
	**Diabetic**	419±38.4 *#	1.6±0.19 *#	126±13 *#	48.6±4.9 *#
	**Diabetic + WP**	284±26 **+**	3.9±0.38 +	83±7.5 +	34±3.8 +
**Day 13**	**Control**	119±13.4	6.4±0.6	52.3±4.8	15.5±1.1
	**Diabetic**	373.6±32 *#	1.45±0.2 *#	89.9±7.8 *#	39.8±3.2 *#
	**Diabetic + WP**	261±25.5 **+**	3.3±0.3 +	67.1±7.2 +	24.6±2.6 +

The levels of blood glucose and insulin in the three groups of mice were monitored on days 1, 4, 7, 10 and 13 post wounding. The levels of ROS and hydroperoxide in the excisional wound tissue collected from the three groups of mice on days 1, 4, 7, 10 and 13 post wounding were also measured. The accumulated data from 10 individual mice from each group are shown. The data were first tested for normality and variance homogeneity prior to any further statistical analysis. The data were normally distributed and are expressed as the mean ± SEM. ^*^P < 0.05, diabetic vs. control; ^**+**^P < 0.05, diabetic + WP vs. control; ^**#**^P < 0.05, diabetic + WP vs. diabetic (ANOVA with Tukey’s post-test).

### The impact of WP supplementation on the levels of proinflammatory cytokines in diabetic mice

**Figure 3:**
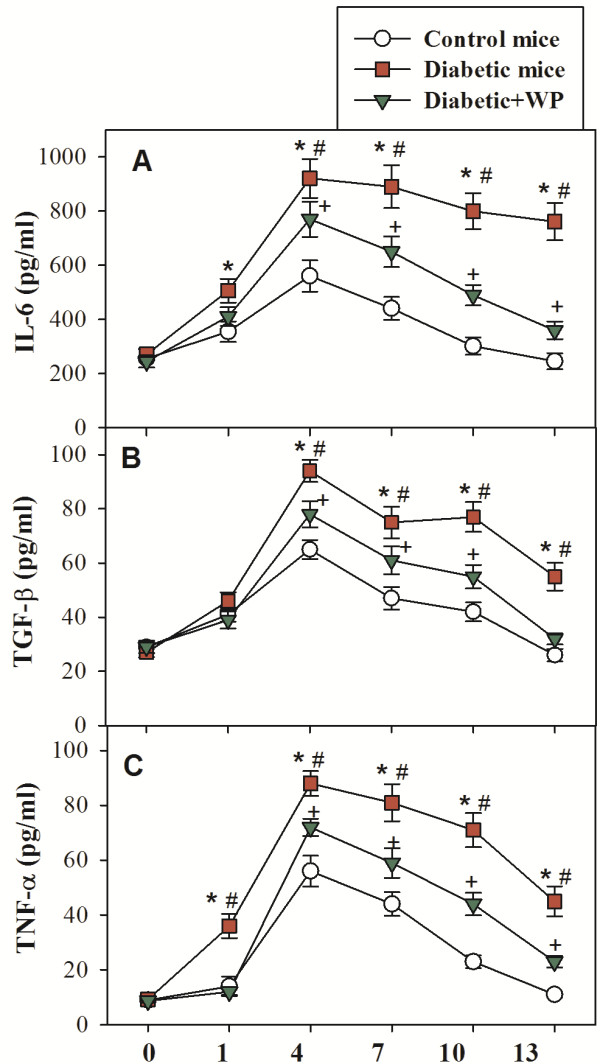
**Effect of WP supplementation on pro-inflammatory cytokines in the wound tissue of diabetic mice.** The levels of IL-6 (**A**), TGF-β (**B**) and TNF-α (**C**) in the excisional wound tissue collected from the three groups of mice on days 1, 4, 7, 10 and 13 post wounding were measured using ELISAs. The cytokines levels from the control skin (day 0, an hour before wounding) were also measured in the three groups of mice. The data were first tested for normality and variance homogeneity prior to any further statistical analysis. The data were normally distributed, are presented as the amount of cytokines (pg) per mg of tissue and are expressed as the mean ± SEM. ^*^P < 0.05, diabetic vs. control; ^**+**^P < 0.05, diabetic + WP vs. control; ^**#**^P < 0.05, diabetic + WP vs. diabetic (ANOVA with Tukey’s post-test).

We monitored the levels of the proinflammatory cytokines (IL-6, TGF-β and TNF-α) that control the process of wound healing in the wounded tissue of the three groups of mice. The accumulated data from 10 individual mice from each group are shown (Figure 3). The diabetic mice had aberrant and significantly higher levels of IL-6 (Figure 3A), TGF-β (Figure 3B) and TNF-α (Figure 3C) 1 through 13 days post injury than the control and WP-treated diabetic mice, suggesting that the wound healing in diabetic mice includes a prolonged proinflammatory phase. By contrast, the supplementation of diabetic mice with WP significantly restored the levels of IL-6, TGF-β and TNF-α.

### Supplementation with WP during diabetes enhanced the expression of β-defensin-1, -2 and -3 in wounded tissue

**Figure 4:**
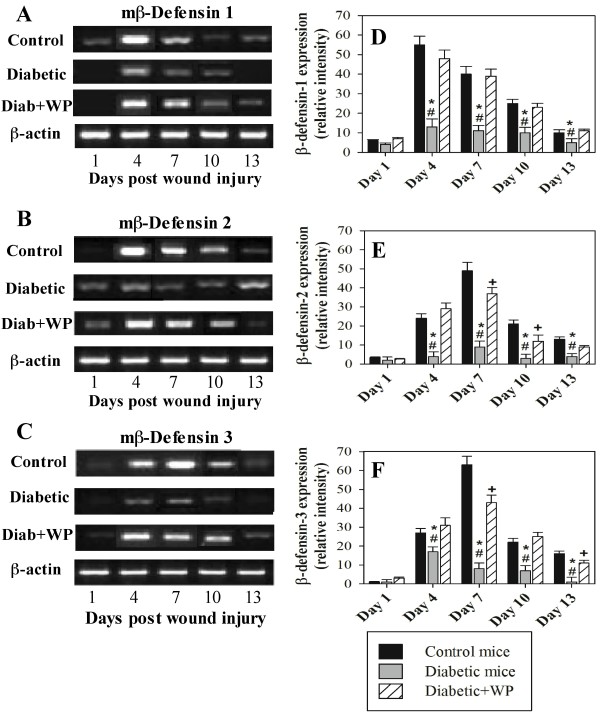
**Effect of WP supplementation on the mRNA expression levels of murine BD-1, 2 and 3 in the wounded tissues.** The mRNA levels of mBD-1, 2 and 3 in the three groups of mice at the indicated time points post wounding were measured using RT-PCR. The expression levels of mBD-1 **(A)**, 2 (**B**) and 3 (**C**) on the indicated number of days post wounding from one representative experiment are shown. The accumulated data on the expression of mBD-1 (**D**), 2 (**E**) and 3 (**F**) from three individual mice from each group are shown, and the results are expressed as the mean ± SEM. ^*^P < 0.05, diabetic vs. control; ^**+**^P < 0.05, diabetic + WP vs. control; ^**#**^P < 0.05, diabetic + WP vs. diabetic.

RT-PCR was performed to measure the mRNA expression levels of BD-1, 2 and 3, which play important roles in the wound healing process, in the excisional wound tissues that were collected from the mice on days 1, 4, 7, 10 and 13 post injuries. The BD-1 (Figure 4A), 2 (Figure 4B) and 3 (Figure 4C) levels from one representative experiment are shown. The accumulated data on the expression levels of BD-1 (Figure 4D), 2 (Figure 4E) and 3 (Figure 4F), which were normalized to the expression of β-actin, from three individual mice from each group are shown. The diabetic mice had significantly lower expression levels of BD-1, 2 and 3 throughout the indicated wound healing time intervals than the control and WP-supplemented diabetic mice. Interestingly, the WP supplementation in the diabetic mice restored the expression levels of BD-1, 2 and 3.

### WP supplementation upregulated the protein levels of BD-1, 2 and 3 in the wounded tissues of diabetic mice

**Figure 5:**
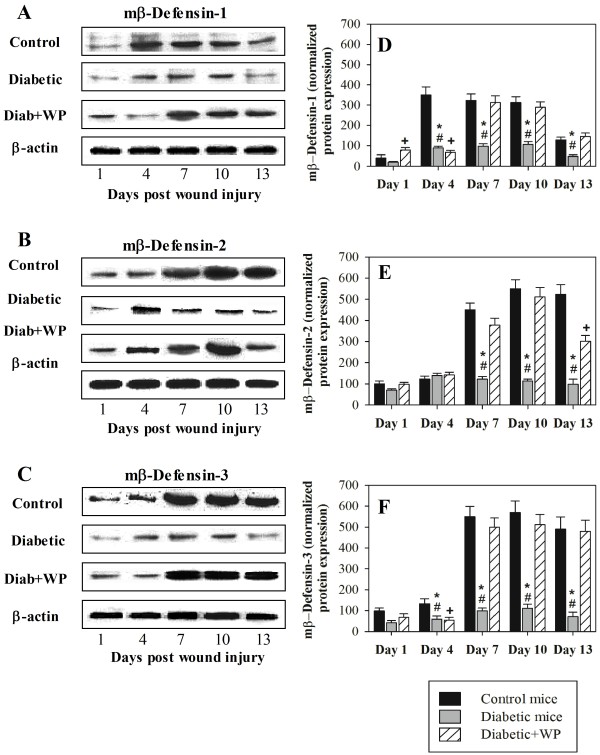
**Effect of WP supplementation on the protein expression levels of murine BD-1, 2 and 3 in the wounded tissues.** The protein levels of mBD-1, 2 and 3 in the 3 groups of mice at the indicated time points post wounding were measured using western blot. The expression levels of mBD-1 (**A**), 2 (**B**) and 3 (**C**) on the indicated days post wounding from one representative experiment are shown. The accumulated data on the expression of mBD-1 (**D**), 2 (**E**) and 3 (**F**) from three individual mice from each group are shown, and the results are expressed as the mean ± SEM. ^*^P < 0.05, diabetic vs. control; ^**+**^P < 0.05, diabetic + WP vs. control; ^**#**^P < 0.05, diabetic + WP vs. diabetic.

To confirm the expression of β-defensins after WP supplementation, western blot analysis measured the protein expression levels of BD-1, 2 and 3 in the excisional wound tissues collected from the 3 groups of mice on days 1, 4, 7, 10 and 13 post injury. The protein expression levels of BD-1 (Figure 5A), 2 (Figure 5B) and 3 (Figure 5C) from one representative experiment are shown. The diabetic mice had longer wound healing times, which were correlated with the aberrant protein expression levels of BD-1, 2 and 3 throughout the measured time intervals of the healing period, than the control and WP-supplemented diabetic mice. The accumulated data on the expression levels of BD-1 (Figure 5D), 2 (Figure 5E) and 3 (Figure 5F), which were normalized to the expression levels of β-actin, from three individual mice from each group are shown. The diabetic mice exhibited significantly lower expression levels of BD-1, 2 and 3 throughout the indicated wound healing time intervals than the control and the WP-supplemented diabetic mice. Interestingly, the WP supplementation of diabetic mice significantly restored the expression levels of BD-1, 2 and 3.

## Discussion

Although the role of nutrition is well established in immune and inflammatory diseases, its role in normal physiological processes, such as cutaneous wound healing, is not well understood [25]. Therefore, several studies have attempted to understand the underlying defects in the wound healing of diabetic patients. We hypothesized that a dietary antioxidant, such as WP supplementation, may improve the delayed wound healing in diabetic patients through the modulation of blood glucose levels, oxidative stress, growth factors and the inflammatory response. The significant reduction in the wound size that was observed after WP treatment was correlated with various histopathological findings: increased epithelization activity, angiogenesis, granulation tissue formation and extracellular matrix remodeling. Collagen not only confers strength and integrity to the tissue matrix but also plays an important role in the homeostasis and epithelialization in the later stages of wound healing [26]. In this study, we found that the macroscopic changes and the rate of wound closure were significantly lower in the WP-supplemented diabetic mice than in the non-supplemented diabetic mice. The accelerated wound closure of the WP-treated diabetic mice may be attributed to increases in GSH synthesis and the cellular antioxidant defense [16]. Because hydroxyproline is a major constituent that is found almost exclusively in collagen, we used its content as an indicator of the amount of collagen type I at the wound sites. We observed that the defective wound repair in the diabetic mice was associated with lower hydroxyproline content; however, the hydroxyproline content increased after the WP treatment. Similarly, Zhang et al. [27] observed that the impaired wound healing in diabetic rats is associated with lower hydroxyproline content. A previous study indicated that the impaired collagen deposition in the acute wounds of type 1 diabetes patients is potentially due to decreased fibroblast proliferation [28]. The enhanced level of hydroxyproline and thus the increased level of collagen likely strengthened the regenerated tissue in the diabetic mice supplemented with WP. Our data revealed that the delayed wound repair observed in the diabetic mice was associated with a significant increase in the blood glucose levels and an obvious decrease in the insulin levels; these effects were reversed by WP supplementation. In previous studies, dietary antioxidant supplementation itself did not affect blood glucose levels [29]. However, the dietary antioxidant supplementation used in this study significantly lowered the blood glucose levels of the diabetic mice even though the blood glucose levels of these diabetic mice were higher than normal levels. In addition, other studies have shown that the addition of whey to meals stimulates insulin release. Moreover, the addition of whey to a lunch meal consisting of mashed potatoes and meatballs reduces the postprandial blood glucose excursion in patients with type 2 diabetes [30]. Oxidative stress is increased by enhancing the rate of ROS production and decreasing the antioxidant defense in diabetic subjects. In this study, we observed that the treatment of diabetic mice with WP lowered the levels of hydroperoxide and free radicals in wounded tissue. Importantly, we observed that the WP treatment improved wound healing in the diabetic mice because the treatment restored the elevated levels of proinflammatory cytokines (IL-6, TGF-β and TNF-α) in the wounded tissues, which limit the prolonged inflammation. This finding demonstrates the mechanism underlying the enhanced immune response and improved wound healing observed in diabetic mice supplemented with WP. These results are corroborated by a previous study that indicated that lactoferrin can regulate the levels of TNF-α and IL-6, which decrease inflammation and mortality [31]. Furthermore, our previous study confirmed these finding [32].

Skin wounding affects the expression of defensins, especially human BD-2 and 3. These skin-derived defensins can effectively promote wound healing due to their antimicrobial effects on pathogens and their stimulatory effects on wound repair cells and immune cells [12,33]. The present study showed correlations between wound closure and the expression of β-defensin after WP supplementation; these correlations show that the supplementation of diabetic mice with WP significantly restored the mRNA and protein expression levels of BD-1, 2 and 3. We therefore hypothesize that it is possible to accelerate the wound closure rate by regulating the β-defensin levels through WP supplementation. This effect can be attributed to lower oxidative stress, as was confirmed by the decreased ROS and hydroperoxide levels detected during the wound healing period. Similar observations suggest that defensins play a role in cell division, immune cell attraction and maturation, cell differentiation and the reorganization of epithelial tissues during the wound healing phases [33]. In conclusion, the results of this study suggest that WP supplementation induces synergetic effects that improve the conditions of diabetes through the regulation of insulin and blood glucose levels, the decrease of oxidative stress and the restoration of the levels of proinflammatory cytokines and β-defensin that accelerate cutaneous wound healing.

## Abbreviations

IL-6: Interleukin-6; mBD: Murine β-Defensin; ROS: Reactive oxygen species; TGF-β: Transforming growth factor-beta; TNF-α: Tumor necrosis factor-alpha; WP: Whey protein

## Competing interests

The author declare no conflicts of interest, state that the manuscript has not been published or submitted elsewhere, state that the work complies with the Ethical Policies of the Journal and state that the work has been conducted under internationally accepted ethical standards after relevant ethical review.
